# Novel insight into theacrine metabolism revealed by transcriptome analysis in bitter tea (Kucha, *Camellia sinensis*)

**DOI:** 10.1038/s41598-020-62859-2

**Published:** 2020-04-14

**Authors:** Songlin Wang, Jiedan Chen, Jianqiang Ma, Jiqiang Jin, Liang Chen, Mingzhe Yao

**Affiliations:** Tea Research Institute of Chinese Academy of Agricultural Sciences, Key Laboratory of Tea Biology and Resources Utilization, Ministry of Agriculture and Rural Affairs, 9 South Meiling Road, Hangzhou Zhejiang, 310008 China

**Keywords:** Plant sciences, Secondary metabolism

## Abstract

Kucha (*Camellia sinensis*) is a kind of unique wild tea resources in southwest China, containing sizeable amounts of theacrine (1,3,7,9-tetramethyluric acid) and having a special bitter taste both in fresh leaves and made tea. Theacrine has good healthy function locally. But the molecular mechanism of theacrine metabolism in Kucha was still unclear. In order to illuminate the biosynthesis and catabolism of theacrine in Kucha plants, three tea cultivars, *C. sinensis* ‘Shangyou Zhongye’ (SY) with low-theacrine, ‘Niedu Kucha 2’ (ND2) with middle-theacrine and, ‘Niedu Kucha 3’ (ND3) with high-theacrine, were used for our research. Purine alkaloid analysis and transcriptome of those samples were performed by High Performance Liquid Chromatography (HPLC) and RNA-Seq, respectively. The related gene expression levels of purine alkaloid were correlated with the content of purine alkaloid, and the results of quantitative real-time (qRT) PCR were also confirmed the reliability of transcriptome. Based on the data, we found that theacrine biosynthesis is a relatively complex process, *N*-methyltransferase (*NMT*) encoded by TEA024443 may catalyze the methylation at 9-*N* position in Kucha plant. Our finding will assist to reveal the molecular mechanism of theacrine biosynthesis, and be applied to selection and breeding of Kucha tea cultivars in the future.

## Introduction

Kucha, a unique kind of tea varieties (*Camellia sinensis*) with bitter taste in both fresh leaves and made tea, was firstly found in Yunnan province, most of which located in the adjoin regions between Guangxi, Guangdong, Hunan and Jiangxi provinces in China^1,2^. Kucha plants are usually semi-arbor trees with large leaves, which are difficult to be discriminated from cultivated tea varieties based on morphology observation. However, the leaves of Kucha are remarkably characterized with strongly bitter taste and special aroma compared to other tea cultivars. In the Kucha growing regions, local residents popularly use its leaves as a medicine to treat wound, inflammatory and diarrhea in daily life.

The tea plant has rich amount of purine alkaloids, such as caffeine and theobromine. Most of tea plants generally contain 25.0 mg/g ~ 45.0 mg/g caffeine, and theobromine has a widely variation range from 0.5 mg/g ~ 46.7 mg/g^3^. A special purine alkaloid, 1,3,7,9-tetramethyluric acid, exists in the Kucha leaves, which was significantly related to the formation of bitter taste^4,5^. Li *et al*. found 2.86% (w/w) of theacrine in dry Kucha leaves, while no detection in tea leaves of Cocoa tea (*C. ptilophylla*) and Longjing tea (*C. sinensis*)^6^. Jin *et al*. identified 36 mg/g of theacrine from a Kucha plant, while null of theacrine in other tea plants such as *C. crassicolumna, C. sinensis* and *C. sinensis* var. *pubilimba*^3^. At present, high purity of theacrine could be separated by HPLC and LC-MS, and obtained by ultrasonic reflux extraction^7,8^. As a unique, naturally occurring purine alkaloid presented in Kucha, more and more studies have been focused on its pharmacology and health function. A few of research showed that theacrine had the benefit effects on anti-inflammation, detoxifying, analgesic and promote sleep^9–11^.

The typical route of caffeine biosynthesis in tea leaves is a ‘xanthosine → 7-methylxantosine → theobromine → caffeine’ pathway^12,13^. Theacrine is an important purine alkaloid converted from caffeine by hydration, oxidation and methylation. In usual, large quantities of purine alkaloids such as caffeine and theobromine, and little theacrine are synthesized in *C. sinensis* plants. Zheng *et al*. used radioactive ^14^C isotope trace method to explore the metabolic pathway of theacrine, which indicate that theacrine was synthesized from caffeine in what is probably a three-step pathway with 1,3,7-methyluric acid acting an intermediate^14^. This was the first demonstration about theacrine biosynthesis pathway in Kucha. However, why the theacrine were highly synthesized and concentrated in Kucha plant? And it still remains unclear about the molecular mechanism of theacrine metabolism in Kucha plant.

In order to determine the molecular mechanism of theacrine metabolism, RNA-Seq was performed using three tea cultivars with different theacrine content. They are Shangyou Zhongye (SY) with minor theacrine, Niedu Kucha 2 (ND2) with medium theacrine and Niedu Kucha 3 (ND3) with high theacrine content. In this study, genes related to theacrine metabolism were explored and identified based on transcriptomes analysis. It will assist to explain the theacrine mechanisms in Kucha plant.

## Results

### Analysis of purine alkaloid content

**Figure 1:**
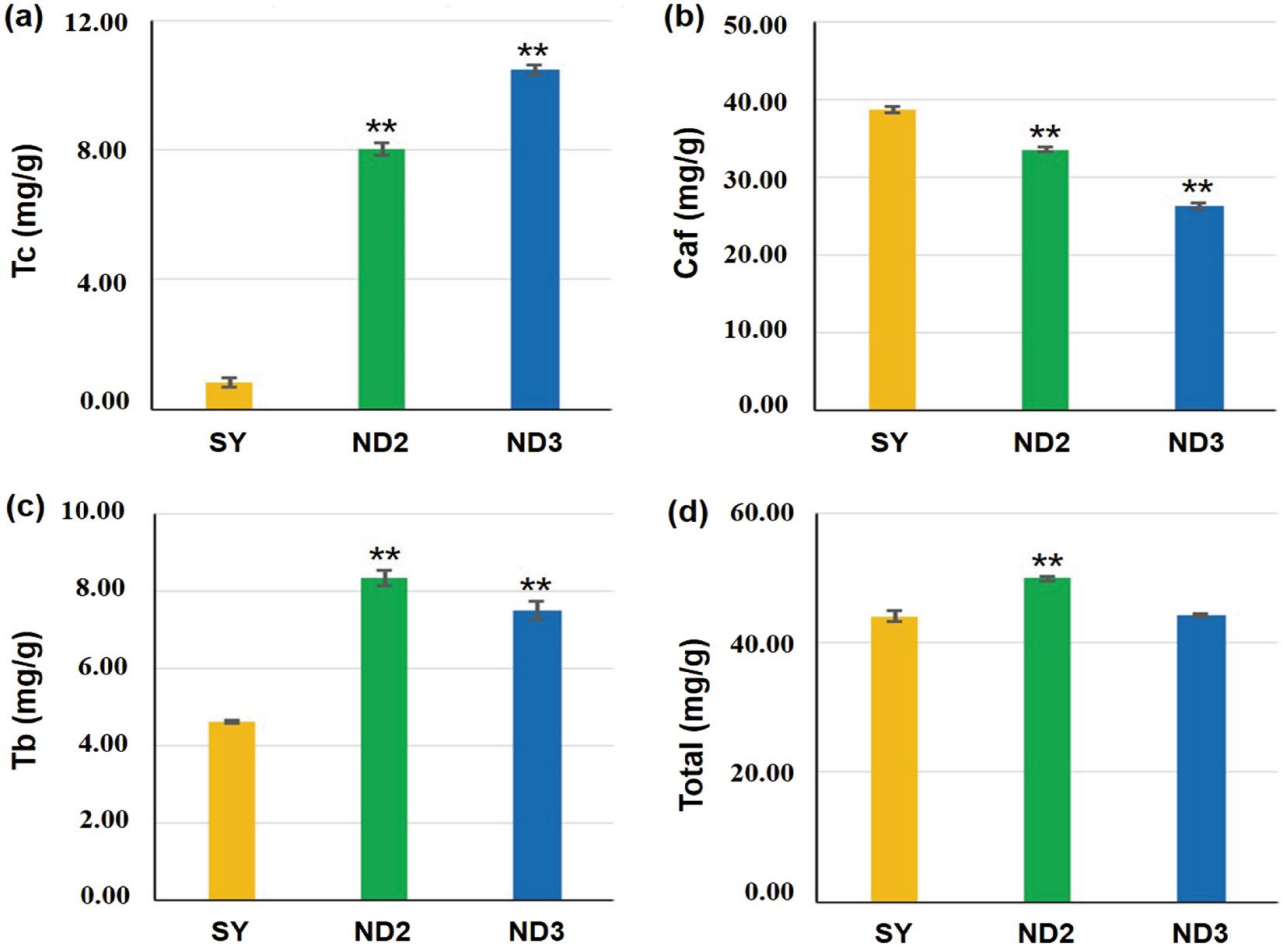
The content of purine alkaloids in SY, ND2 and ND3. (**a**) Tc: theacrine content, (**b**) Caf: caffeine content, (**c**) Tb: Theobromine content, (**d**) Total: total purine alkaloid content. *The significance of the difference compounds between two comparison groups (SY vs ND2, SY vs ND3) is shown as **P <0.01.

The total purine alkaloids were relatively stable in tested samples, they were no significant difference between SY and ND3, especially. However, the significant differences of theacrine and caffeine were found between them. The theacrine content were 8.04 mg/g and 10.45 mg/g in ND2 and ND3 respectively, significant higher than SY (0.83 mg/g). While the variation of caffeine content was opposite to theacrine between the tested samples (Fig. 1). The caffeine was estimated 33.47 mg/g, 26.24 mg/g in ND2 and ND3 respectively, they were considerably lower than SY (38.62 mg/g). It may due to caffeine was the precursor of the theacrine synthesis, which was partly converted to theacrine in Kucha. Moreover, the content of theobromine in ND2 (8.34 mg/g) and in ND3 (7.49 mg/g) was considerably higher than those in SY (4.60 mg/g).

### Sequencing analysis and reference genome alignment

Three cDNA libraries of ND2, ND3 and SY were constructed and sequenced. Totally, 18.87 Gb high-quality reads were obtained from paired-end reads, with a single read length of ~150 bp. A total of 41.28, 42.84 and 41.33 million high-quality reads were generated from ND2, ND3 and SY, respectively. Q20 percentage values were over 96.65%, while the Q30 percentage value were more than 92.56%, and the GC content was ranged from 49.10% ~ 49.29% (Table 1). The average mapped ratio of three samples to the genome was 92.28%. These results showed that the obtained transcriptomic data with relatively high comparison to the reference genome could be used for the further research.

**Table 1:** Quality evaluation of sequencing data in ND2, ND3, SY.

Sample	High-quality reads	Clean base (Gb)	Q20 (%)	Q30 (%)	Error (%)	GC content (%)	Total mapped reads	Total Mapped ratio (%)
ND2	41,278,692	6.19	96.65	92.56	0.012	49.24	36,056,312	91.42
ND3	42,839,490	6.42	97.75	94.40	0.006	49.10	31,241,850	91.66
SY	41,331,604	6.20	97.65	94.17	0.005	49.29	30,857,847	93.77

### Analysis of different expression genes (DEGs)

**Figure 2:**
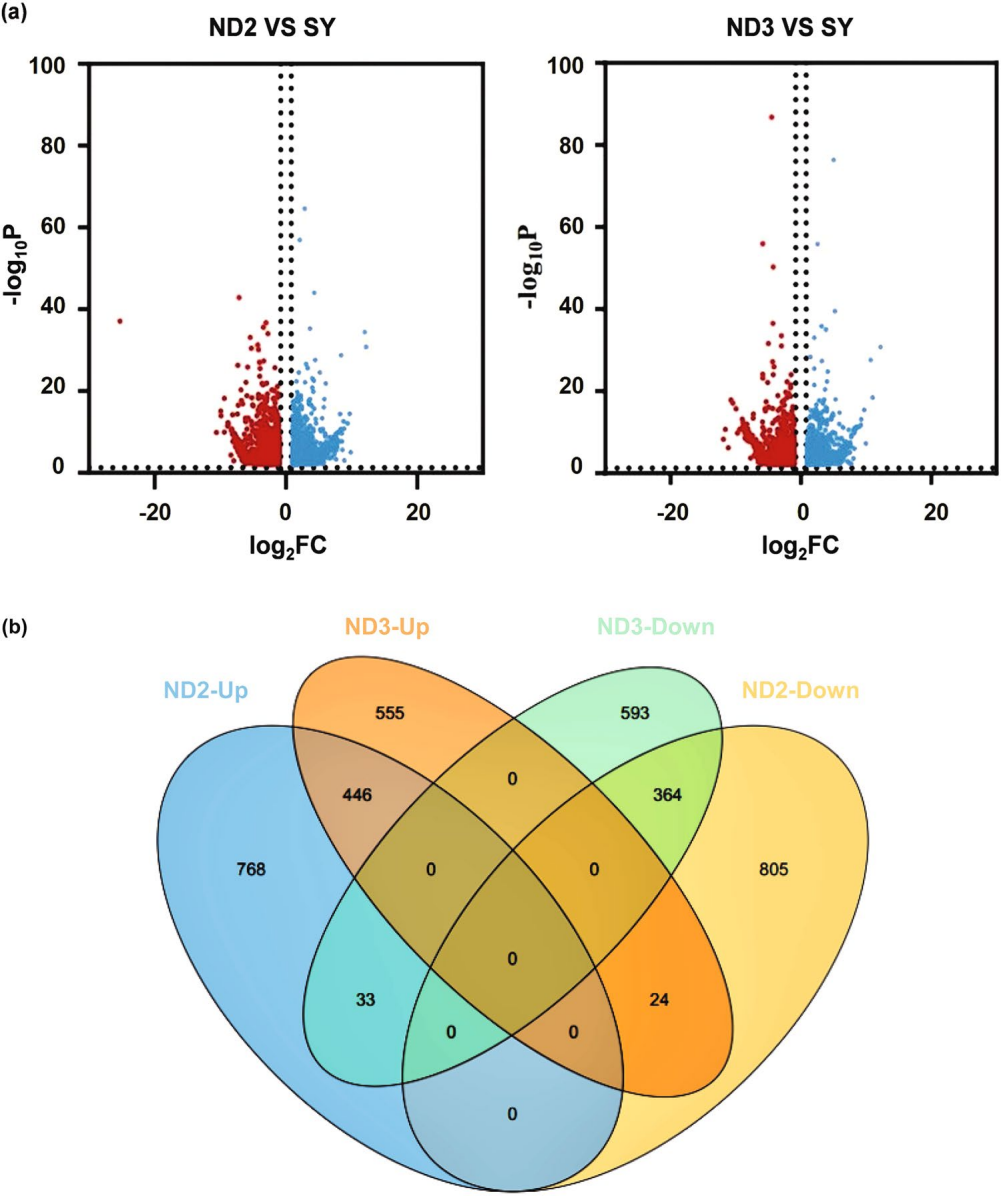
(**a**) Volcano plots of DEGs. (**b**) Venn diagram of DEGs: up-regulated DEGs and down-regulated DEGs.

A total of 3,588 DEGs were detected among three Kucha cultivars. In ND2 vs SY, 2,440 DEGs were noted, of which 1,247 were upregulated and 1,193 were downregulated. And 2,015 DEGs were identified in ND3 vs SY, of which 1025 were upregulated and 990 were downregulated. While 810 differentially co-expressed genes (446 up-regulated and 364 down-regulated) were identified between two group of ND2 vs SY and ND3 vs SY (Fig. 2a,b). These genes may reveal the difference in metabolic pathway of purine alkaloid between high- and low- theacrine tea plant.

### GO enrichment analysis of DEGs

GO (Gene Ontology) is an international standard database for gene functional classification, which contains three categories, molecular function (MF), cellular component (CC) and biological process (BP)^15^. In the MF category, the molecular function, the catalytic activity and the binding were the top three enriched GO terms of two groups. In the CC category, DEGs were enriched in terms of the cellular component, the cell and the cell part. And in the BP category, the biological process, the metabolic process and the cellular process were the top terms.

**Figure 3:**
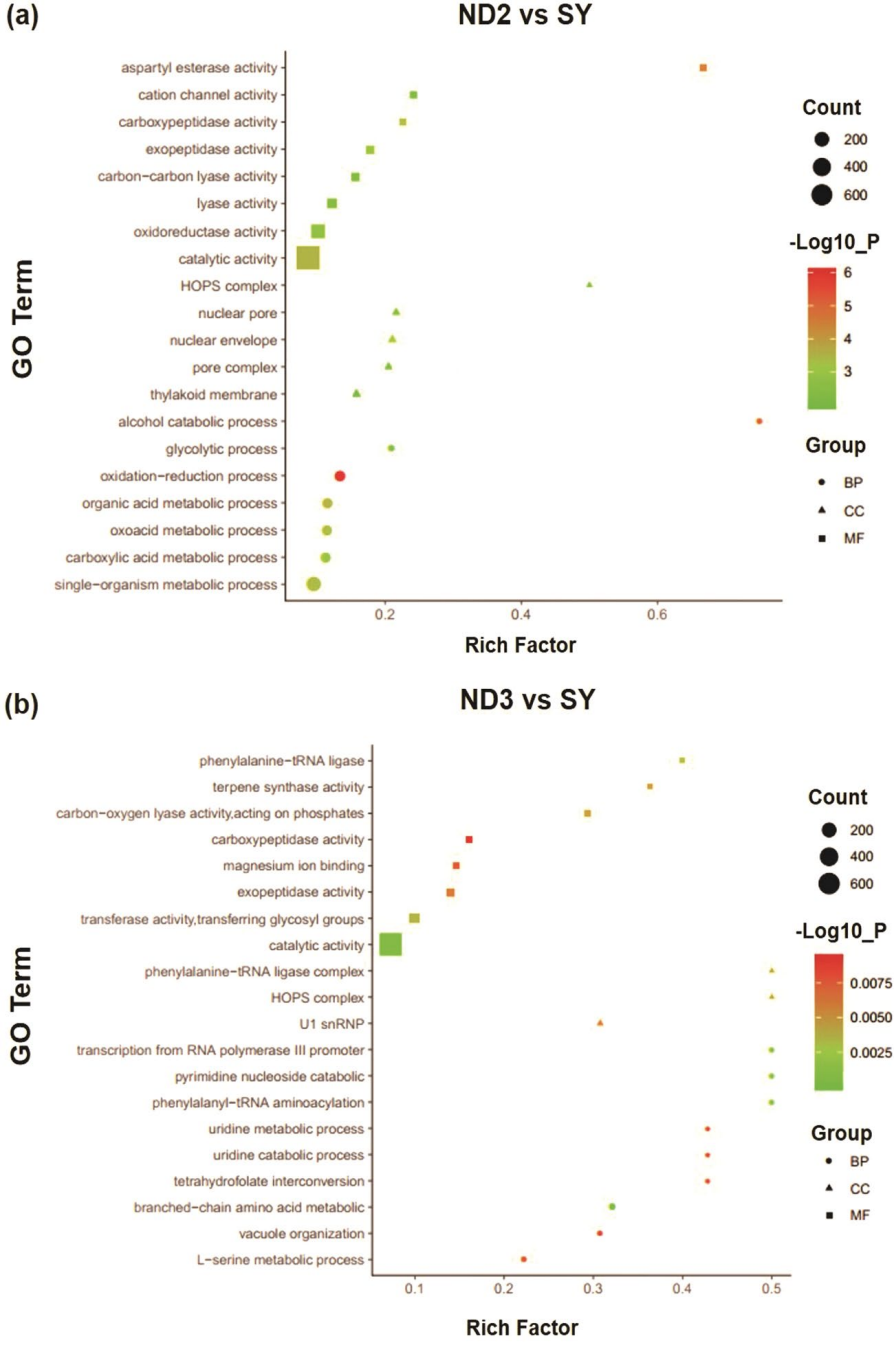
The top 20 enriched GO terms of ND2 vs SY (**a**) and ND3 vs SY (**b**). The rich factor is the ratio of the number of DEGs and all annotated genes. BP: Biological process, CC: Cellular component, MF: Molecular function.

In ND2 vs SY, 1327, 150, and 1465 DEGs were significant enriched into 106, 15 and 48 subcategories of BP, CC and MF, respectively. And in ND3 vs SY, 492, 295, and 840 DEGs were significant enriched into 41, 8 and 25 subcategories of BP, CC and MF, respectively. Moreover, the terms of the methylation, the *S*-adenosylmethionine-dependent methyltransferase activity and the *N-*methyltransferase activity were enriched in 23, 11 and 7 genes in two groups, which may play a key role in purine alkaloid biosynthesis. The top 20 enriched terms of GO classification are shown in Fig. 3.

### KEGG enrichment analysis of DEGs

**Figure 4:**
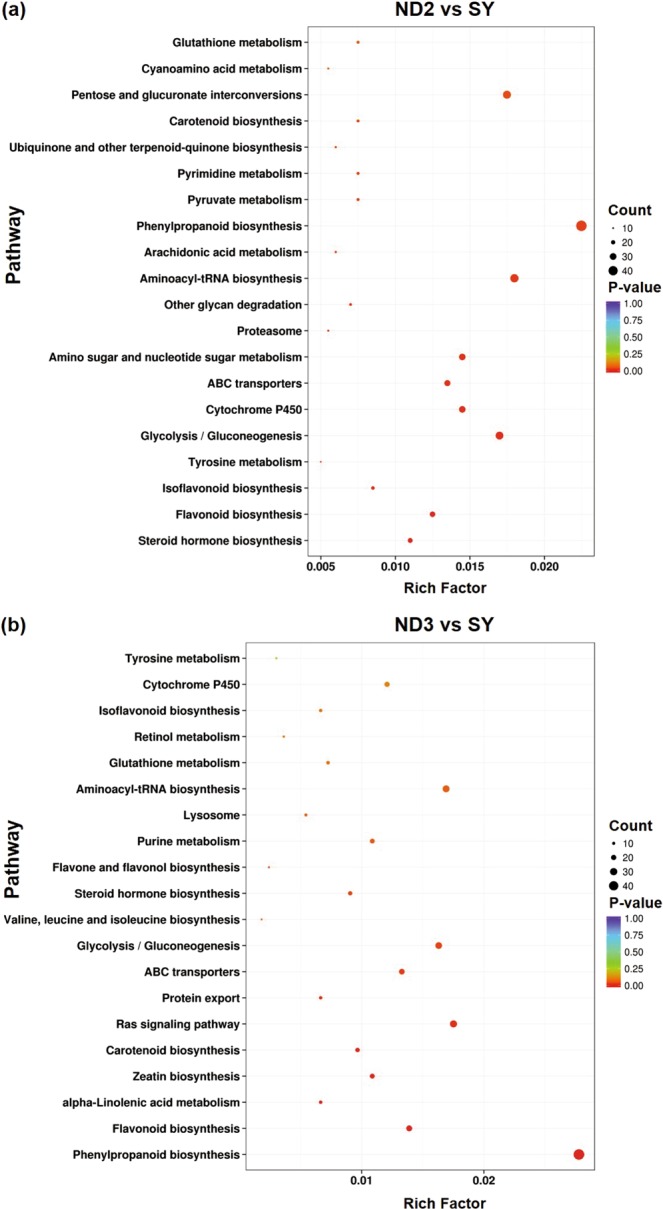
The top 20 KEGG pathways of ND2 vs SY (**a**) and ND3 vs SY (**b**). The rich factor is the ratio of the number of DEGs and all annotated genes.

To explore whether the related genes of metabolism in Kucha were enriched, we constructed the KEGG pathway analyses by the obtained unigenes which corresponding reference pathway^16^. Results showed that 192 and 182 metabolic pathways in ND2 vs SY and ND3 vs SY group, respectively. These pathways contain flavonoid biosynthesis, phenylpropanoid biosynthesis, carotenoid biosynthesis, ABC transporters and steroid hormone biosynthesis were significant enriched in both group (Fig. 4). Moreover, a certain amount of DEGs were noted in secondary metabolite biosynthesis pathway, such as purine metabolism, flavone and flavonol biosynthesis, tyrosine metabolism, tropane, piperidine and pyridine alkaloid biosynthesis and indole alkaloid biosynthesis.

### Gene expressions in purine alkaloid metabolism

**Figure 5:**
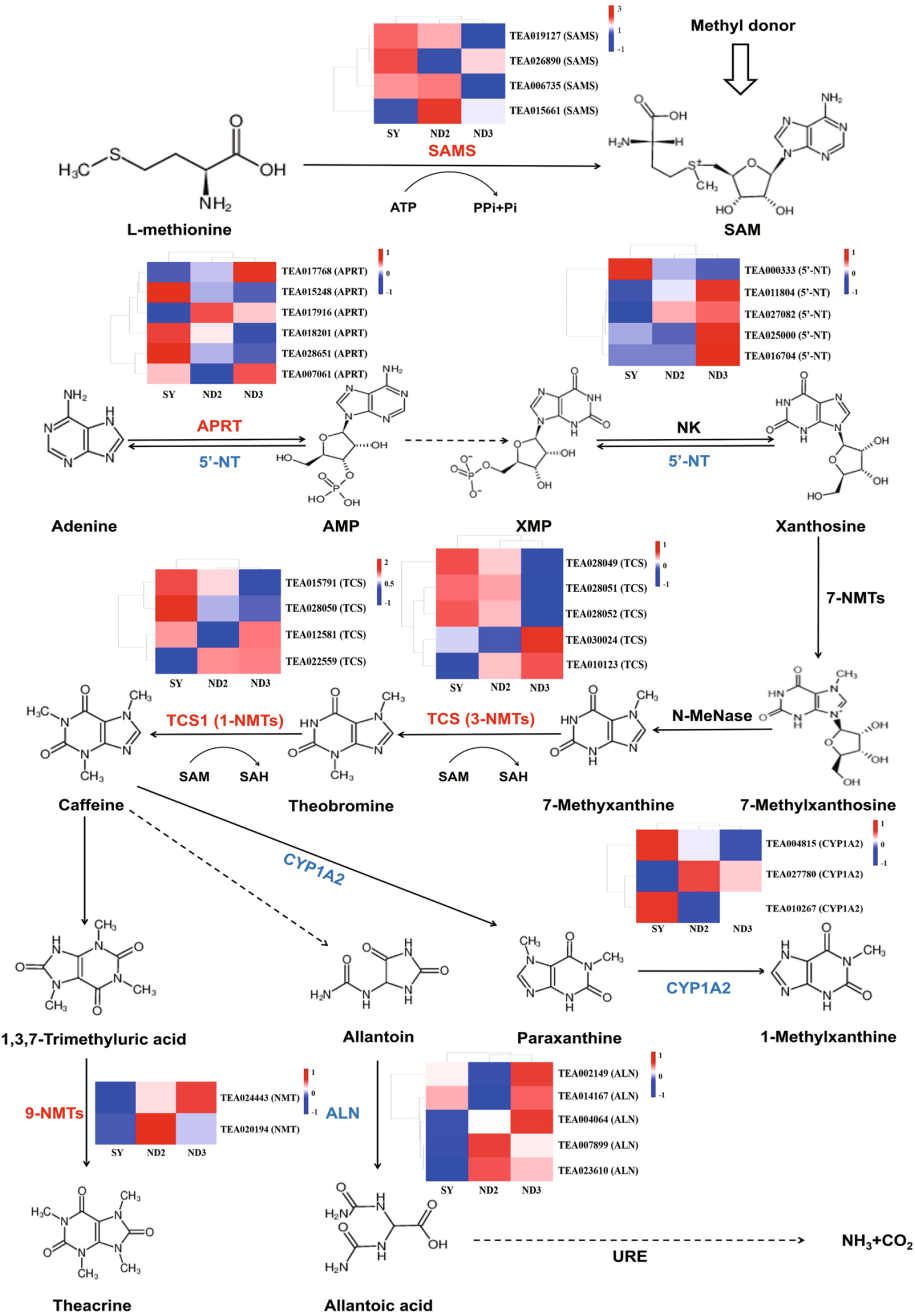
Purine alkaloid metabolic pathway. Dotted line indicates that some metabolites have been omitted. The column chart shown that the gene expression of three tea samples. The red genes play a synthetic role and the blue represent degradation. *SAMS, *S*-adenosylmethionine synthetase, APRT, adenine phosphoribosyltransferase; NK, nucleoside kinase; 5′-NT, 5′-nucleotidase; 3-NMT and 1-NMT, tea caffeine synthase (TCS); N-MeNase, *N*-methyl nucleosidase; CYP1A2, cytochrome P450 family 1 subfamily apolypeptide 2; ALN, allantoinase; URE, urease.

Caffeine metabolism was the main pathway of purine alkaloids in *C. sinensis*^17^. Theobromine is the intermediate product in caffeine synthesis process, while theacrine and theophylline are in caffeine degradation process. The pathway of “7-Methyxanthosine → 7-Methyxanthine → Theobromine → Caffeine → 1,3,7-Trimethyluric acid → Theacrine” was the only process for theacrine synthesis at present. The purine salvage process is “Adenosine →AMP → XMP → Xanthosine”. In addition to the process of “caffeine → theacrine”, we also annotated other three caffeine degradation pathway in KEGG database, the final degradation products in this pathway are “NH_3_ + CO_2_”, “7-Methyluric acid” and “1-Methyluric acid”, respectively (Fig. 5). The identification of those pathway can help us to further explain the metabolism of purine alkaloids in tea plant.

The expression patterns of key genes in theacrine synthesis such as *TCS, SAMS, APRT, NMT* were conduced to ascertain the reason of high concentration of theacrine in Kucha plant (Fig. 5). TEA015248 and TEA028651 (encoded adenine phosphoribosyltransferase, *APRT*) in the adenine donor synthesis, TEA015791, TEA028050, and TEA028052 (encoded tea caffeine synthase, *TCS*) were highly expressed in SY with high caffeine concentration. TEA006735 and TEA015661 (encoded *S*-adenosylmethionine synthetase, *SAMS*) in the methyl donor synthesis were up-regulated in ND2 which contains the most purine alkaloid among three samples. The expression levels of TEA024443 (encoded *N*-methyltransferase, *NMT*) were up-regulated in ND2 and ND3 which had a highly content of theacrine compare with SY. We also identified the key genes of purine alkaloid degradation, TEA027082 and TEA011804 (encoded 5′- nucleotidase, *5*′*- NT*) which involved the degradation pathway of “AMP → Adenosine” and “Guanosine→GMP” were up-regulated in ND2 and ND3 compared with SY. TEA002149 (encoded allantoinase, *ALN*) was highly expressed in ND3 with high theacrine concentration. From the KEGG enrichment analysis, 5 and 6 DEGs which annotated in cytochrome P450 family were obtained in SY vs ND2, and SY vs ND3, respectively. TEA010267 and TEA004815 (encoded cytochrome P450 family 1 subfamily A polypeptide 2, *CYP1A2*) were highly expressed in high caffeine concentration sample.

Pearson correlation coefficient was used to evaluate the correlation between the gene expression level and purine alkaloid content (Table 2). The results shown that the expression patterns of TEA028049 (*TCS*), TEA015248 (*APR*T) and TEA000333 (*5*′*-NT*) were negatively correlated with the content of theacrine (P < 0.05). Meanwhile, TEA028050 (*TCS*) and TEA028052 (*TCS*) were positively correlate with caffeine content (P < 0.05), especially TEA018201 (*APRT*) presents the significantly positive correlation (P < 0.01). It was speculated that the proteins encoded by these genes were involved in the regulation of theacrine and caffeine content in the Kucha plants.

**Table 2:** The correlation coefficient of purine alkaloid and gene expression levels.

Gene name	Gene ID	Correlation Coefficient		
		TC	CAF	TB
*NMT*	TEA024443	0.983	−0.986	0.799
*NMT*	TEA020194	0.224	−0.548	−0.232
*SAMS*	TEA019127	−0.860	0.984	−0.544
*SAMS*	TEA006735	−0.648	0.872	−0.243
*SAMS*	TEA026890	−0.581	0.262	−0.882
*SAMS*	TEA015661	0.339	0.009	0.721
*TCS*	TEA028049	−1.000*	0.947	−0.885
*TCS*	TEA028050	−0.994	0.971	−0.840
*TCS*	TEA028051	−0.931	1.000*	−0.672
*TCS*	TEA028052	−0.924	0.999*	−0.659
*TCS*	TEA030024	0.992	−0.887	0.945
*TCS*	TEA010123	0.933	−1.000**	0.677
*TCS*	TEA012581	0.885	−0.992	0.587
*TCS*	TEA015791	−0.906	0.997	−0.625
*TCS*	TEA022559	0.987	−0.871	0.956
*APRT*	TEA017916	0.749	−0.473	0.965
*APRT*	TEA007061	0.019	−0.365	−0.426
*APRT*	TEA015248	−0.999*	0.955	−0.871
*APRT*	TEA028651	−0.973	0.833	−0.974
*APRT*	TEA018201	−0.936	1.000**	−0.370
*APRT*	TEA017768	0.837	−0.975	−0.508
*5*′*-NT*	TEA011804	0.993	−0.890	0.942
*5*′*-NT*	TEA025000	0.668	−0.885	0.269
*5*′*-NT*	TEA027082	0.484	−0.151	0.822
*5*′*-NT*	TEA016705	0.694	−0.901	0.302
*5*′*NT*	TEA000333	−0.999*	−0.916	−0.660
*CYP1A2*	TEA010267	−0.939	0.762	−0.994
*CYP1A2*	TEA027780	0.998*	−0.956	0.870
*CYP1A2*	TEA004815	−0.992	0.975	−0.832
*ALN*	TEA002149	0.323	−0.631	−0.130
*ALN*	TEA007899	0.685	−0.390	−0.994
*ALN*	TEA023610	0.838	−0.597	−0.893
*ALN*	TEA014167	0.993	−0.303	0.601
*ALN*	TEA004064	0.972	−0.994	0.285
*URE*	TEA020308	0.700	−0.409	0.944

### qRT-PCR analysis of the DEGs

**Figure 6:**
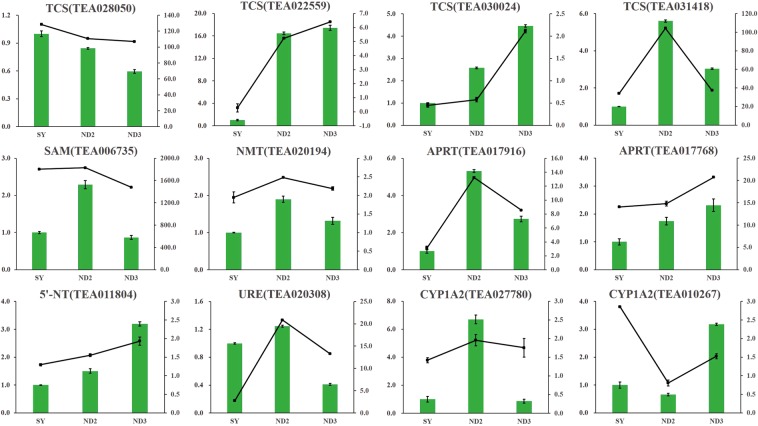
qRT-PCR verification of the selected DEGs involved in caffeine and theacrine metabolic pathway. *The histogram represent the relative expression of the genes in three sample (ND2, ND3 and SY) by qRT-PCR, and the expression pattern (FPKM) of the genes obtained from RNA-Seq was indicated by the trend line.

To further confirm the reliability of the gene expression levels, 12 genes which were functionally related to purine alkaloid metabolism were selected for verification by quantitative real-time (qRT) PCR analysis, and their primers are listed in Table S1. Results shown that, 12 genes, except for TEA027780 (*CYP1A2*) and TEA010267 (*CYP1A2*), were generally consistent with RNA-Seq which further indicated the reliability of the transcriptome date (Fig. 6).

## Discussion

As a kind of special tea germplasm, the classification of Kucha still remains being under discussion. In Zhang Hongda classification method, Kucha was defined as *C. sinensis* var. *kucha* Zhang et Wang^18^; Du *et al*. defined Kucha as *C. sinensis* var. *kuiea* Du et Li based on methods of chemical classification and digital taxonomy^19^; Min studied the morphological characteristics of Kucha cultivars, indicated that Kucha should be classified into *C. sinensis* var. *assamica*, not be defined as a new tea variety^20^; Chen *et al*. considered that Kucha should be classified into *C. sinensis* var. *assamica*, according to the systematic study of tea resources and the comprehensive investigation of tea plants in original place^21^; Wang *et al*. determined biochemical components of 24 Kucha resources, indicated that Kucha germplasms were similar to *C. sinensis* var*. assamica* in terms of catechin composition which was reflected the degree of tea plant evolution^22^. For Kucha classification, further study should be focused on genomic level by comparing Kucha plant with other tea species.

Meanwhile, the exploitation and utilization of bitter tea resources are also an important problem faced by breeders. Jianghua Kucha (a bitter tea) has been studied and utilized deeply which was widely promoted in the area of broken black tea at Hunan province, because of its suitable for black tea^23–25^. In our study, High level of purine alkaloids in Kucha cultivars is conducive to the processing of high-quality black tea, and it also provides a new way to develop product of natural purine alkaloid and multi-purpose exploitation of Kucha.

Theacrine is an advantage purine alkaloid second only to caffeine in Kucha plant generally, which affect quality and flavor of Kucha products significantly. Previous studies indicated that the theacrine varied in different Kucha cultivars, and less affected by environments^22^. Ye *et al*. found that the content of theacrine was stable with season change in young leaves of Kucha. While the theacrine was significantly higher in immature leaves than that in mature leaves and aged leaves^4^. At present, metabolic pathway of purine alkaloid and the related genes of biosynthesis have been basically determined which lay the foundation for our research of theacrine metabolism^26,27^. In our study, RNA-Seq was used to discovered the transcriptome of three tea cultivars, DEGs involved in purine alkaloid metabolism were discovered in two comparison groups ND2 vs SY and ND3 vs SY. Contents of theacrine and caffeine among three cultivars were obviously different, theacrine in ND2 and ND3 was higher than SY, while the caffeine content was lower.

KEGG pathway analysis indicated that purine alkaloid metabolism pathway plays significant roles in Kucha metabolism, and a number of key genes related to theacrine and caffeine biosynthesis were selected from these DEGs. Theacrine biosynthesis is catalyzed by a series of enzyme, including *S*-adenosylmethionine synthetase (SAMS), tea caffeine synthase (TCS), *N*-methyltransferase (NMTs) and Adenine phosphoribosyltransferase (APRT). In general, SAMS is the key enzyme in purine alkaloid metabolism pathway, of which *S*-adenosylmethionine act as methyl donor in theacrine and other purine alkaloids biosynthesis^28^. Our results show that the gene expression levels of *SAMS* were up-regulated in two groups, which the total amount of purine alkaloids was higher than that of control variety. TCS is a class of *N*-methyltransferase that catalyzes the methylation of *N*-3 (theobromine synthase) and *N*-1 (caffeine synthase) in tea plant. Previous studies point that *TCS1* plays a crucial role for methylation of xanthosine at 1-*N* position in caffeine biosynthesis, while *TCS1* alleles with low transcription level or encoded proteins with no TCS activity^29^. TEA015791, encoded TCS1, was down-regulated in ND2 and ND3, it may be the reason that why the lower content of caffeine in two Kucha samples. But no research indicated that TCS has the ability to catalyzed methylation at 9-*N* position. It still needs the further research that whether TCS involved in the final step of theacrine biosynthesis. TEA024443, encoded NMTs, was highly expression in high-theacrine sample which was positively correlated with the content. *NMT* may play a significant role in theacrine synthesis which dominant the pathway of “1,3,7-trimethyl xanthine (caffeine)→1,3,7-trimethyluric acid→1,3,7,9-tetramethyluric acid (theacrine)”, we speculated that *N*-methyltransferase encoded by this gene responsible for catalyze the methylation at 9-*N* position in theacrine synthesis. Meanwhile, the expressions of related gene in purine alkaloid degradation pathway was probably correlated with the decreased of purine alkaloid in Kucha samples. TEA007899 and TEA023610, encoded allantoinase (ALN), and TEA020308, encoded urease (URE), were all highly expression in ND2 and ND3 with low-caffeine Kucha plant, which involved the final degradation step of “Allantoin→Allantoic acid → →NH_3_ + CO_2_”.

Caffeine is seemed as a precursor of theacrine, its biosynthesis pathway is very clear. And the key genes involved in caffeine metabolism have been identified, which can provide reference for the further study of theacrine^29–31^. However, until now few studies have been reported on molecular mechanism of theacrine biosynthesis in Kucha cultivars. In this study, we proposed an NMT, encoded by TEA024443, was likely the key enzyme involving in the final synthesis process of theacrine. Next, the gene of TEA024443 should be focused on verification and testing its function in theacrine biosynthesis. Further, we will construct the tea genetic population with Kucha cultivars as parents, key genes involved in theacrine metabolism were identified and analyzed by density genetic map and QTL mapping. It will make it possible to breeding new tea cultivar which is rich in theacrine yet contains low caffeine.

To sum up, our study provided a new insight to illuminating theacrine molecular metabolism in Kucha plants, and it may help to the breeding improvement of Kucha plant in subsequent studies.

## Methods

### Plant materials

Three tea cultivars were used as plant materials in this study, including high-theacrine cultivar ‘Niedu Kucha 3′ (ND3), medium-theacrine ‘Niedu Kucha 2′ (ND2) and low-theacrine ‘Shangyou Zhongye’ (SY) as a control. The former two are Kucha accessions from the same local population of tea landrace named ‘Niedu Kucha’, the latter one is local cultivated tea clone. All of them were collected from the same region located in the southwest part of Jiangxi province. And they are currently preserved in National Germplasm Hangzhou Tea Repository, in the Tea Research Institute of the Chinese Academy of Agricultural Sciences at Hangzhou city, Zhejiang province in China. Leaves of two Kucha plants are tasted very bitter, while that of the controlled cultivar is not bitter. Fresh healthy leaves were picked on the two terminal leaf position from ND2, ND3 and SY respectively, quickly frozen in liquid nitrogen firstly, and then stored at −80 °C for RNA extraction. And the young shoots with “two leaves and a bud” collected from three cultivars were fixed with hot air at 120 °C for 5 min and then dried to constant weight at 80 °C. These dried samples were used to detect content of purine alkaloid such as theacrine, caffeine and theobromine using HPLC method. All experiments had three biological replicates.

### Identification of purine alkaloids

Purine alkaloids such as theacrine, caffeine and theobromine were detected by HPLC methods described in a previous reported method^32^. A Waters High Performance Liquid Chromatography (HPLC) instrument was used to detect the compounds in this experiment. A Waters C_18_ column (5 μm, 4.6 cm × 250 mm) was performed at a flow rate of 1.0 mL/min with the column temperature of 35 °C. 1% formic acid was used as mobile phase A, 100% acetonitrile as mobile phase B, 10 μL sample liquid was injected and analyzed at 280 nm, and the elution time was 45 min.

### RNA extraction and library construction

EASY-spin Plus Complex Plant RNA kit (Aidlab Biotechnologies Co., Beijing, China) was used to extract total RNA. The integrity and quantity of total RNA were estimated by NanoDrop ultraviolet spectrophotometer (Thermo, Waltham, MA) and agarose gel electrophoresis. cDNA libraries established by the standard mRNA-Seq Library Prep Kit, and the library quality was assessed by using the Agilent 2100 Bioanalyzer (Agilent, Palo Alto, CA). The cDNA libraries were sequenced by the Illumina HiSeq platform, performed by Personal Biotechnology Co., Ltd. (Beijing, China).

### Gene expression analysis

High-quality reads were evaluated and acquired from the raw data, which need to filter out the low-quality sequence, adaptor sequences, duplicated sequences and ambiguous (with the ratio of N > 5%). The clean reads from all the samples were mapped to *C*. *sinensis* ‘Shuchazao’ genome by HISAT software with default parameters and counted by feature counts^33,34^.

Gene Ontology (GO) and Kyoto Encyclopedia of Genes and Genomes (KEGG) annotation were downloaded from http://tpia.teaplant.org/. The DEGs (differentially expressed genes) among tea samples (three biological replicates per group) were identified by DESeq. 2. Genes with a FDR (false discovery rate) ≤ 0.05 and an absolute fold change value ≥ 1.0 were identified as DEGs^35,36^.

### Validation of quantitative real-time PCR

To confirm the accuracy of RNA-Seq and DEG analyses, 12 DEGs associated with purine alkaloid biosynthesis pathway were selected for qRT-PCR. The primers of specific genes for qRT-PCR were designed by Primer Premier 5, and the primer pair sequences were listed in Table S1. First-strand cDNA (10-fold dilution) synthesis from total RNA was operated by using FastKing RT kit (with gDNase) (TianGen Biotech Co, Beijing). qRT-PCR was performed in ABI7500 (Applied Biosystems Inc, US), and the GAPDH (glyceraldehyde 3-phosphate dehydrogenase) gene were utilized as the internal reference. The total volume of the qRT-PCR reaction is 20 μl which contained 7.4 μL distilled H_2_O, 0.6 μL primer each pair, 10 μL 2x FastFire qPCR PreMix, 0.4 μL 50x Rox Reference Dye△ and 1 μL diluted cDNA. The cycling profile was 95 °C, 60 s; 40 cycles of 95 °C, 5 s, and 60 °C, 32 s in 96-well optical reaction plates. The relative gene expression was calculated though the 2^−△△Ct^ method^37^. Each sample was examined in three technical replicates.

### Statistical analysis

Three biological replicates of sequencing data and purine alkaloid components were used for calculating the mean value and the standard deviation (SD) based the Duncan’s multiple range tests. SPSS 18.0 was used to analyze the correlations of gene expression and purine alkaloid concentrations via Pearson correlation. The volcano figure was produced with Prism 7.0 software. The double coordinate figures and heat maps were draw with R package.

## Supplementary information

Supplementary Information.
