# Supplementary material for: Novel insight into theacrine metabolism revealed by transcriptome analysis in bitter tea (Kucha, Camellia sinensis)
Source: Sci Rep. 2020 Apr 14;10:6286. doi: 10.1038/s41598-020-62859-2 (PMC7156766; doi:10.1038/s41598-020-62859-2)
Supplement: Supplementary file 1 — Supplementary Information. [file 41598_2020_62859_MOESM1_ESM.docx]

**Supplementary Information**

**Novel insight into theacrine metabolism revealed by transcriptome analysis in bitter tea (Kucha,** ***Camellia sinensis*)**

**Songlin Wang, Jiedan Chen, Jianqiang Ma, Jiqiang Jin, Liang Chen* & Mingzhe Yao***

* Correspondence: Liang Chen: [liangchen@tricaas.com](mailto:liangchen@tricaas.com), +86-0571-86652835;

MingZhe Yao: [yaomz@tricaas.com](mailto:yaomz@tricaas.com), +86-0571-86650444

Tea Research Institute of Chinese Academy of Agricultural Sciences, Key Laboratory of Tea Biology and Resources Utilization, Ministry of Agriculture and Rural Affairs, 9 South Meiling Road, Hangzhou, Zhejiang 310008, China

**Supplementary Table**

**Table S1.** The primer pair sequences used for qRT-PCR

| **Gene name** | **Gene ID** | **Forward primer (5’-3’)** | **Reverse primer (5’-3’)** |
| --- | --- | --- | --- |
| *TCS* | TEA028050 | AGTTTCTTGTTATGTGATGGGAGTA | GGAACCACCTCTTCGGATTT |
|  | TEA022559 | TGGAGGTGAAAGAAGCGTTGT | ACTGGCATTGTCATTGAGGC |
|  | TEA030024 | AGTGGCCTCAATGAAAATGC | CACTGTGAACGTGGTTGGAC |
|  | TEA031418 | ATGGATTAGAGGGGAAAAGT | TGATTTCATGTCCAAACTGG |
| *URE* | TEA020308 | CTCCCGATAGTAGATGACAGAA | CCTCAATAAAGTGATAGTGGC |
| *5’-NT* | TEA011804 | TGAGTCTGGGATGCTTCTTA | CAACTCTTATTCAGGCTCTTTA |
| *APRT* | TEA017768 | GTATGGAGGCTGAGGTGGTC | AATGTTCTATTTGTCGTGGC |
|  | TEA017916 | GGTGAACGAGCCGTGGTAAT | ACCTGACAGCGTCTGAAAGA |
| *SAMS* | TEA006735 | CGAGCCATTGTCAGTGTTTG | GCTTTGAGTTCTTCGCCATC |
| *NMT* | TEA020194 | TTGCCGTTGCGGAGTGGAGT | TGCCGTCTTCTTGCTTCTTTGGTT |
| *CYP1A2* | TEA010267 | TGCGGGCACTGATACTACAT | GTGACGCCTCAACCCTTCCA |
|  | TEA027780 | ACGACGACAAGGGAACCAAG | TCGCTGCATAGGCTCACCAT |
